# Optogenetic protein clustering through fluorescent protein tagging and extension of CRY2

**DOI:** 10.1038/s41467-017-00060-2

**Published:** 2017-06-23

**Authors:** Hyerim Park, Na Yeon Kim, Sangkyu Lee, Nury Kim, Jihoon Kim, Won Do Heo

**Affiliations:** 1 Department of Biological Sciences, Korea Advanced Institute of Science and Technology (KAIST), Daejeon, 34141 Republic of Korea; 2 Center for Cognition and Sociality, Institute for Basic Science (IBS), Daejeon, 34141 Republic of Korea

## Abstract

Protein homo-oligomerization is an important molecular mechanism in many biological processes. Therefore, the ability to control protein homo-oligomerization allows the manipulation and interrogation of numerous cellular events. To achieve this, cryptochrome 2 (CRY2) from *Arabidopsis thaliana* has been recently utilized for blue light-dependent spatiotemporal control of protein homo-oligomerization. However, limited knowledge on molecular characteristics of CRY2 obscures its widespread applications. Here, we identify important determinants for efficient cryptochrome 2 clustering and introduce a new CRY2 module, named ‘‘CRY2clust’’, to induce rapid and efficient homo-oligomerization of target proteins by employing diverse fluorescent proteins and an extremely short peptide. Furthermore, we demonstrate advancement and versatility of CRY2clust by comparing against previously reported optogenetic tools. Our work not only expands the optogenetic clustering toolbox but also provides a guideline for designing CRY2-based new optogenetic modules.

## Abstract (web summary)

Cryptochrome 2 (CRY2) from *A. thaliana* can be used to control light-dependent protein homo-oligomerization, but the molecular mechanism of CRY2 clustering is not known, limiting its application. Here the authors identify determinants of CRY2 clustering and engineer fusion partners to modulate clustering efficiency.

## Introduction

Protein homo-oligomerization is among the essential molecular mechanisms that control various signaling proteins, such as membrane receptors, kinases, and transcription factors^1, 2^. Therefore, a synthetic tool for controlling protein homo-oligomerization would be invaluable for manipulating numerous biological events. The most widely used approaches for conditionally inducing protein homo-oligomerization have been chemically induced homo-association systems^3^. However, the limitations of the chemical compound, such as low reversibility and poor spatiotemporal resolution, restrict its use of studying the dynamic nature of protein activity. One approach for addressing these drawbacks was an introduction of cryptochrome 2 (CRY2) from *Arabidopsis thaliana* to control target protein oligomerization at specific times and spaces through exploitation of the light-induced homo-oligomerization property of CRY2^4–9^. Yet, accumulating evidence suggests that robust clustering of CRY2 was only available under certain conditions, depending on CRY2 protein concentration and kind of tagging proteins^9–11^. In addition, due to unknown CRY2 structure, although the CRY2 clustering tool has been exploited by different groups, it still remained unsettled, which factors are critical determinants and how clustering of CRY2-fused target proteins can be efficiently generated.

In this study, we sought to provide an insight into how fusion proteins or tags influence the efficiency of CRY2-based oligomerization, thereby developing a novel CRY2 module to achieve robust and efficient oligomerization of target proteins in response to blue light. By exploiting various fluorescence proteins (FPs), consisting of a common backbone structure but different oligomeric states, we demonstrated that quaternary structure of a protein fused to CRY2 influenced the efficiency of CRY2 clustering. In addition, simply by tagging with newly identified short peptide, we engineered an outstanding CRY2 clustering system (CRY2clust), which is capable of efficiently regulating fine cellular signaling in living cells in response to light.

## Results

### Effect of quaternary structures of FPs on CRY2 clustering

**Fig. 1:**
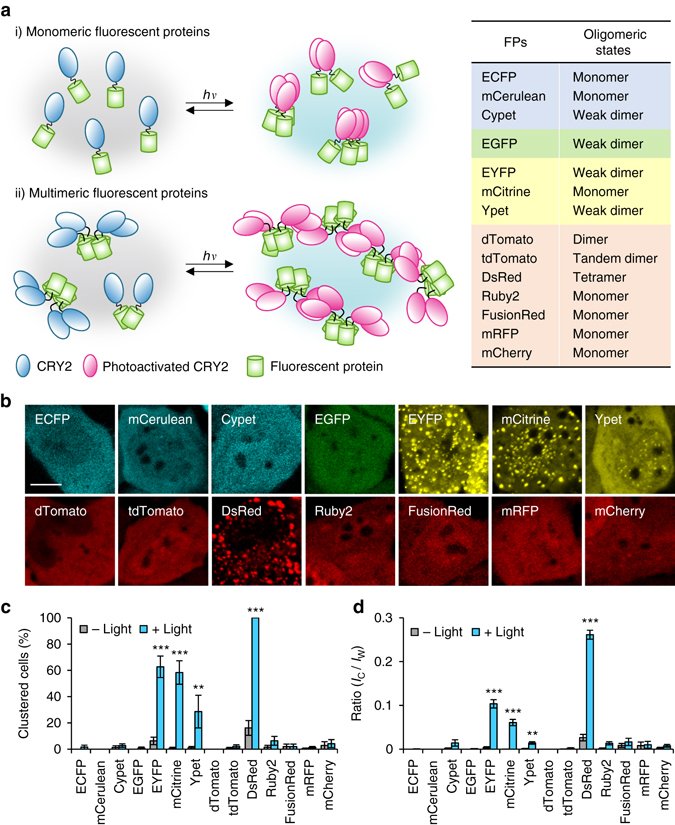
Effect of quaternary structures of FP tags on the efficiency of light-induced CRY2 clustering. **a** Schematic diagram for visualizing relationships between quaternary structures of FPs and efficiency of CRY2 clustering. *Right*, lists of characteristics of FPs used in this research. **b** Fluorescence images of HeLa cells transfected with expression plasmids for each FP-labeled CRY2PHR. Scale bar, 10 μm. **c** Quantification of the percentage of clustered cells. **P = 2.03 × 10^–5^; ***P = 4.11 × 10^–17^ (EYFP), 5.44 × 10^–12^ (mCitrine), 4.87 × 10^–46^ (DsRed) by Student’s two-tailed *t*-test. **d** Quantification of the cluster ratio in cells containing clusters. Total cluster intensity (*I*_C_) was divided by the total fluorescence (*I*_W_) of the whole-cell. Values are expressed as means ± s.e.m. (*n* = 122, 81, 127, 100, 188, 193, 223, 163, 174, 113, 140, 171, 187, 142 cells, three independent experiments). **P = 8.36 × 10^–6^; ***P = 6.74 × 10^–20^ (EYFP), 1.66 × 10^–13^ (mCitrine), 4.87 × 10^–46^ (DsRed) by Student’s two-tailed *t*-test

Given previous studies that light-induced CRY2 clustering seems to depend on target proteins fused to CRY2, we initially hypothesized that oligomeric states of a protein fused to CRY2 might be a key determinant for CRY2 clustering (Fig. 1a). To systematically analyze relationships between quaternary structures of tagging proteins and the efficiency of CRY2 clustering, we used structurally well-characterized FPs^12^, which have a same backbone structure but different oligomeric states. Fourteen different FP candidates (Fig. 1a) were conjugated on photolyase homology region of CRY2 (CRY2PHR) [amino acids 1–498]. In dark state, most CRY2PHR-conjugated FPs appeared to be distributed throughout the cytoplasm. Upon light illumination, CRY2PHR proteins conjugated with dimeric EYFP and Ypet, and tetrameric DsRed remarkably induced cluster formation^12, 13^ (Fig. 1b; Supplementary Fig. 1 and Supplementary Movie 1). To compare efficiencies of cluster formation among different CRY2PHR-conjugated FPs, we analyzed the percentage of clustered cells (Fig. 1c) and cluster ratios (Fig. 1d). Again, EYFP, Ypet and DsRed showed high cluster formation efficiency out of the transfected population and exhibited an enhanced cluster ratio compared to other tagged FPs. Although in overall, the result implied the positive correlation of clustering efficiency with the extent of multivalency of fused FP, we realized that monomeric mCitrine attached to CRY2PHR also underwent light-induced homo-oligomerization. Given that oligomeric states of FPs were mostly characterized under in vitro conditions^12^ and that some FPs reported to be monomer form oligomers under physiological conditions^14, 15^, our results also raise the possibility that some FPs adopt different quaternary structures in the intracellular environment.

Moreover, we examine the effect of FP conjugation site on CRY2PHR clustering. Intriguingly, conjugation of FP at C-terminal CRY2 (CRY2PHR-FPs) exhibited higher clustering efficiency than conjugation at N-terminal CRY2 (FPs-CRY2PHR) except for DsRed conjugation, which demonstrated high clustering ratio in both cases (Supplementary Fig. 2). This implies that as well as the quaternary structure of tags for CRY2, there might be an additional factor for influencing efficient CRY2 clustering.

### Superior CRY2 clustering by conjugating of short peptides

**Fig. 2:**
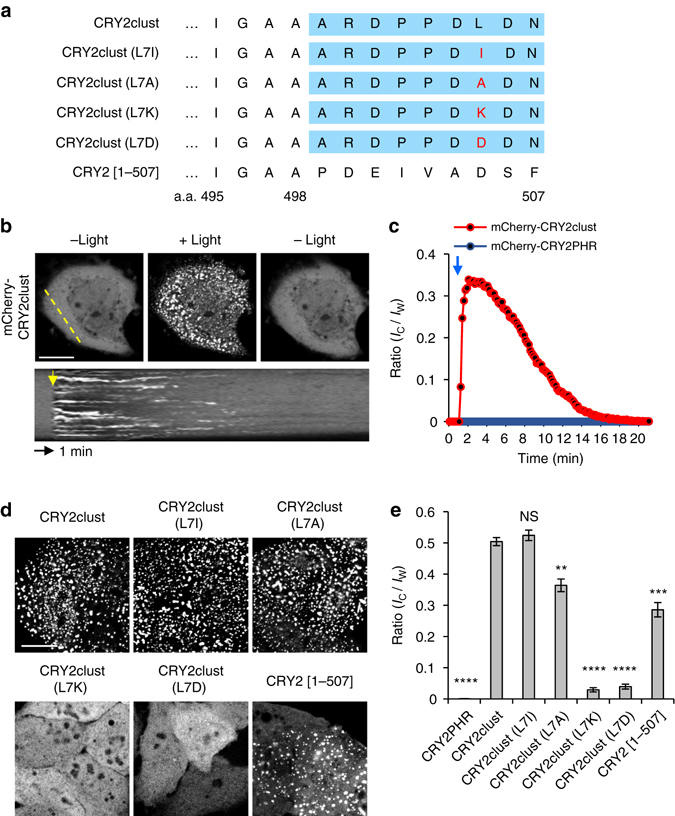
Enhancement of light-induced CRY2 clustering by a C-terminal extension. **a** Sequences of CRY2 variants extended at the C-terminus of CRY2PHR [a.a. 1–498] with the nine residues of a new peptide (*blue box*) or those of native CRY2 [a.a. 1–507]. Mutated residues are shown in *red*. **b** Fluorescence images of a cell showing reversible clustering of mCherry-CRY2clust. *Bottom*, kymograph corresponding to the *yellow line* in the *top left* image. **c** Graph of time-lapse results showing changes in cluster ratio. Both *yellow* and *blue arrows* in **b**, **c** indicate illumination time points. **d** Fluorescence images of cells expressing each of the indicated mCherry-labeled CRY2 variants, upon light stimulation. Cells were illuminated with light for 5 min at 20 s intervals. **e** Quantification of cluster ratio. Values are expressed as means ± s.e.m. (*n* *=* 72, 99, 91, 76, 98, 88, 97 cells, three independent experiments). **P = 3.45 × 10^–8^; ***P = 5.57 × 10^–14^, ****P = 9.42 × 10^–62^ (CRY2PHR), 1.32 × 10^–69^ (CRY2clust(L7K)), 9.97 × 10^–70^ (CRY2clust(L7D)) by Student’s two-tailed *t*-test. Scale bars, 20 μm

In a course of generating different CRY2PHRs with various FPs, we serendipitously discovered a very short (nine residues) peptide, which substantially enhanced light-induced CRY2 clustering (Fig. 2a). Under our experimental conditions, little cluster formation was observed in cells expressing mCherry-CRY2PHR (Figs. 1 and 2c; Supplementary Figs. 1 and 2), whereas C-terminal extension of CRY2PHR with the 9-residue peptide, termed CRY2clust showed robust cluster formation (within seconds) after a pulse of blue light that was reversible upon termination of light stimulation (Figs. 2b, c; Supplementary Movie 2).

To investigate the critical determinants of the peptide in CRY2clust for clustering enhancement, we first examined an effect of conjugation site of the peptide on CRY2PHR. Similar with results from FP conjugation, only a fusion of the peptide on C-terminal CRY2PHR induced the robust clustering (Supplementary Fig. 3). Then, a length of the peptide was questioned that we performed serial deletion of the C-terminus of the CRY2clust, and expressed each variant in HeLa cells. Whereas no variants showed clusters in the dark state, under light illumination, CRY2clust (∆8–9 a.a.) and (∆9 a.a.) variants exhibited robust clustering similar to that of CRY2clust in almost all cells (Supplementary Fig. 4). The result suggests that first seven amino acids are necessary for the enhanced CRY2 clustering property.

According to our observation on enhanced clustering by the oligomeric FPs, we examined whether CRY2clust could affect multivalency of CRY2 in the dark. To test this, we visualized co-translocation of CRY2PHR variants such as CRY2PHR and CRY2clust to the plasma membrane (PM) or mitochondria (Mito) by orthogonal inputs; rapamycin, and light (Supplementary Fig. 5a). Upon rapamycin treatment in the dark, FKBP-FRB heterodimerization led to translocation of FKBP-fused CRY2PHR variants to the PM or Mito and predicted that if CRY2PHR variants have homo-association property, they would co-translocate to the compartment along with FKBP-fused CRY2PHR variants. As a result, we barely observed co-translocation of mCherry-CRY2PHR variants, but light stimulation remarkably accumulated mCherry-CRY2PHR variants at the PM or Mito. Notably, light-dependent recruitment of CRY2clust to the PM was much more efficient than that of CRY2PHR, consistent with data showing superior clustering of CRY2clust (Supplementary Fig. 5). Therefore, the results indicate low possibility of CRY2clust to be a multimer in the intracellular environment in the dark state.

Since the result suggest that first seven amino acids are necessary for the enhancement of CRY2 clustering, to further elucidate the mechanism of CRY2clust for enhancement of clustering efficiency, we speculated the amino acid property of Leu at position 7 of C-terminus of CRY2clust might have a role in robust CRY2 clustering. We replaced Leu with different amino acids: hydrophobic Ile (L7I) and Ala (L7A), polar uncharged Asn (L7N) and Gln (L7Q), basic Lys (L7K) and acidic Asp (L7D) (Fig. 2a). When exchanged to hydrophobic residues (L7I, L7A), no immense changes on efficient CRY2 clustering was observed. In contrast, substitution of Leu with uncharged polar amino acids (L7N, L7Q) and charged residues (L7K, L7D) significantly disrupted cluster formation (Fig. 2d, e; Supplementary Fig. 6). Interestingly, we found out that level of hydrophobicity of substitutes^16^ at position 7 is highly correlated with clustering efficiency (Supplementary Fig. 6b). From the mutational analysis along with serial truncation experiment (Supplementary Figs. 4 and 6), we conclude that hydrophobicity at position 7 of C-terminus of CRY2clust is a critical determinant for superior CRY2 clustering.

### Identifying a role for intrinsic C-terminus on CRY2 function

Although CRY2 is known to consist of two domains, PHR and CCE (cryptochrome C-terminal extension) domain (Supplementary Fig. 7a), in most optogenetic studies using CRY2, PHR domain has only been widely used for homo-interactions with itself. However, full-length CRY2 also forms nuclear bodies that appear to generate clusters in plants in response to blue light^17, 18^. Since C-terminal extension of CRY2PHR with the peptide enhanced the clustering efficiency of CRY2, we wondered if extending the C-terminus of CRY2PHR with native CRY2 sequence would have a similar role in the clustering ability of CRY2. To test this, we extended CRY2PHR by serially adding additional amino acids, and labeled it with mCherry. Surprisingly, only one variant containing a 9-amino-acid extension of PHR region at C-terminal, mCherry-CRY2 [1–507], greatly increased the efficiency of light-dependent CRY2 clustering (Fig. 2d, e; Supplementary Fig. 7b, c).

Since hydrophobicity was the important factor of CRY2clust for CRY2 clustering, the aromatic hydrophobic property of Phe, which is located at position 507 of CRY2, was hypothesized as an essential factor. For examination, we replaced the Phe to different amino acid such as alternative aromatic hydrophobic Trp (F507W) and Tyr (F507Y), aliphatic hydrophobic Leu (F507L) and Ala (F507A), uncharged polar Thr (F507T) and charged His (F507H), and Asp (F507D) (Supplementary Fig. 8). Unlike other substitutions, we observed the replacement with alternative aromatic hydrophobic residue (F507Y or F507W) showed CRY2 clustering at the comparable level of wild-type CRY2 [1–507] or even more efficiently, respectively. Particularly, although His contains aromatic ring but low hydrophobicity, substitution with His (F507H) prevented the ability of CRY2 for light-induced clustering, supporting the idea of hydrophobicity importance for enhanced CRY2 clustering. Similar to the results of CRY2clust characterization, we could find out that hydrophobicity of amino acid at position 507 is highly correlated with the extent of cluster formation. Altogether, we suggest a high potential of both CRY2clust and CRY2 [1–507] have a similar mechanism for CRY2 clustering.

### Characteristics comparison of CRY2-based clustering systems

**Fig. 3:**
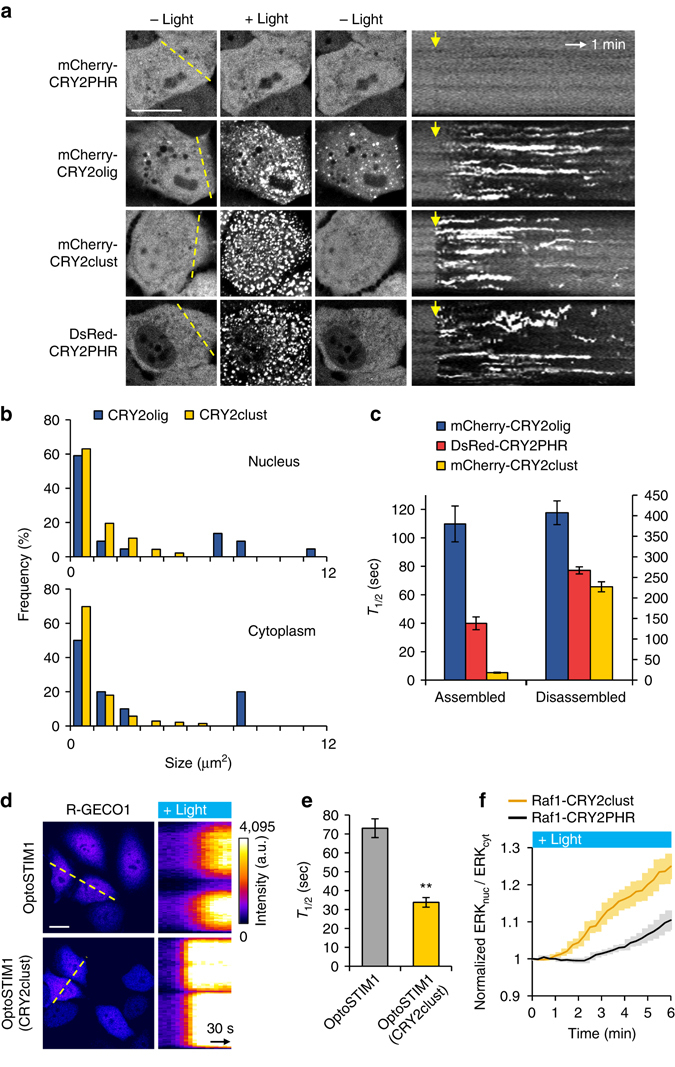
Comparison of characteristics of CRY2-based clustering modules. **a** Fluorescence images of cells expressing each of the indicated constructs following exposure to blue light for 1 s. *Right*, kymographs corresponding to *yellow lines* in *left* images. *Yellow arrows* indicate illumination time points. **b** Size distribution of clusters in the nucleus (*top*) and in the cytoplasm (*bottom*) in cells expressing either CRY2olig or CRY2clust. **c** Graph showing time to reach half-maximal and basal cluster ratio (*T*_1/2_) for assembly and disassembly, respectively (*n* = 29, 35, 34 cells). **d** Fluorescence images of R-GECO1 in cells coexpressing either OptoSTIM1 or OptoSTIM1 (CRY2clust). *Right*, kymographs corresponding to *yellow lines* in *left* images. **e** Graph showing time to reach half-maximal R-GECO1 fluorescence after light illumination in cells expressing the indicated construct (*n* = 62, 75 cells). **P = 3.65 × 10^–10^ by Student’s two-tailed *t*-test. **f** The normalized nuclear/cytoplasmic ERK2-EGFP ratio upon light illumination on cells expressing the indicated optogenetic Raf1 module (*n* = 32, 48 cells). Values are expressed as means ± s.e.m. Scale bars, 20 μm

In addition to our findings, two optogenetic systems for CRY2 clustering enhancements were reported: membrane-bound CRY2PHR^11^ and CRY2olig containing an E490G mutation^9^. In the case of membrane-tethered CRY2PHR, membrane localization of CRY2PHR allows a higher local concentration of CRY2 to promote cluster formation. However, ineffective clustering of cytoplasmic CRY2PHR remains as a barrier in this system. To further investigate the dynamics of cytoplasmic CRY2 clustering, we compared the enhancement in the clustering property of cytoplasmic CRY2PHR achieved in the current study with that of the previously described CRY2olig. The transfected HeLa cells with each of the CRY2 clustering systems were exposed to blue light for 1 s. All CRY2 clustering systems, excluding mCherry-labeled wild-type CRY2PHR, underwent remarkable light-induced clustering in the nucleus and in the cytoplasm, and these clusters disassembled upon withdrawal of the light (Fig. 3a). Whereas the size distribution of CRY2clust clusters was similar in both the nucleus and the cytoplasm, CRY2olig clusters showed exceptional accumulation at a certain location in nucleus immediately after light exposure (Fig. 3b; Supplementary Fig. 9a), as described previously^9^. The cluster pattern in the nucleus of CRY2olig was similar to a pattern of nuclear speckles, which are enriched in pre-mRNA splicing factors than did other subcompartments^19, 20^. To determine whether nuclear localized CRY2 clusters are co-localized with nuclear speckles, we coexpressed EGFP-labeled SC35 splicing factor, a nuclear speckle marker^21^, with mCherry-labeled either CRY2olig or CRY2clust. Using structured illumination microscopy (SIM), we confirmed that CRY2olig clusters showed a higher level of co-localization with EGFP-SC35 compared to clusters of CRY2clust (Supplementary Fig. 9b, c).

Next, when we compared the kinetics of cluster assembly and disassembly among CRY2 clustering tools, CRY2clust showed much faster dynamics than others (Fig. 3a, c; Supplementary Fig. 9a). As reported previously^9^, kinetics and efficiency for cluster assembly of CRY2 correlated with its intracellular concentration in all CRY2 clustering modules. Interestingly, the clustering of CRY2clust appeared to be less affected by its intracellular concentrations and occurred rapidly and efficiently at even low expression levels, compare to other modules (Supplementary Figs. 10 and 11). This could be due to a higher sensitivity of CRY2clust for efficient cluster generation on light stimulation (Supplementary Fig. 12).

### Improvement of CRY2-based optogenetic system with CRY2clust

For versatile CRY2clust applications, a target conjugation sites, either N or C terminus of CRY2clust, should not be restricted that mCherry was conjugated on either site. Neither sites of mCherry conjugation disturbed the clustering ability of CRY2clust (Supplementary Fig. 13), illustrating the potential capability of application onto diverse targets for function regulation. As an example, C-terminus of CRY2PHR in OptoSTIM1, a Ca^2+^-modulating optogenetic system^7^, was extended with the 9-residue peptide (OptoSTIM1(CRY2clust)) and expressed in HeLa cells. Under blue light exposure, OptoSTIM1 proteins harboring CRY2PHR variants (CRY2PHR and CRY2clust) induced notable increases in R-GECO1 fluorescence, a red fluorescent Ca^2+^ sensor^22^ (Fig. 3d; Supplementary Fig. 14). Consistent with the rapid clustering kinetics of CRY2clust, OptoSTIM1 (CRY2clust) caused two-fold faster changes in intracellular Ca^2+^ levels than the original OptoSTIM1 did (Fig. 3d, e; Supplementary Fig. 14). To further verify its versatility, we applied CRY2clust to another optogenetic system for controlling protein kinase activity^23^, Raf1-CRY2PHR. Either mCherry-labeled Raf1-CRY2PHR or Raf1-CRY2clust was coexpressed with ERK2-EGFP in HeLa cells. Light illumination caused translocation of ERK2 from the cytoplasm to nucleus, indicating the activation of Raf1 signaling in both systems by the light. Similar to the result with OptoSTIM1 case, faster translocation of ERK2 by applying CRY2clust than the original Raf1 system was observed (Fig. 3f). This phenomenon was further confirmed by FusionRed-ERK KTR sensor^24^, by additionally showing faster deactivation kinetics with Raf1-CRY2clust (Supplementary Fig. 15a). To note, we observed no significant difference in basal ERK activation levels between Raf1-CRY2PHR and Raf1-CRY2clust (Supplementary Fig. 15b), consistent with our observation that CRY2clust is unlikely to change the degree of homo-association of CRY2PHR in the dark. Therefore, these results strongly support that CRY2clust is generally applicable to control a variety of signaling events with higher temporal resolution but no significant change of basal activity.

## Discussion

In this study, we examined how fusion proteins or tags can influence the efficiency of CRY2 clustering and identified critical determinants for robust CRY2 clustering to achieve efficient control of target protein functions. For this issue, by using various FPs with a common backbone structure but different oligomeric states, we demonstrated that different oligomeric properties of a protein fused to CRY2 influenced the efficiency of CRY2 clustering. Intriguingly, we realized that threshold level of oligomeric states of fused proteins for determining robust CRY2 clustering is between weak dimer to tetramer. The efficiency of CRY2 clustering was also highly affected by conjugation site, implying the existence of alternative mechanism as well as the quaternary structure of tagged protein to determine clustering efficiency of CRY2. One possible explanation could be stabilization of photoactivated CRY2 structure via coupled folding mechanism of CRY2PHR and its C-terminal tag^17^. Although clustering efficiency was greatly enhanced by tagging multimeric proteins such as DsRed, a direct conjugation of such a multimeric protein to CRY2 raises a possibility of perturbing biological functions of protein of interest even in dark state. Altogether, we believe that these findings will provide a guideline to design future experiments using CRY2 for specific purposes and evidence for explaining how CRY2 form clusters.

While examining CRY2 characteristics of light-dependent homo-oligomerization, we developed a superior CRY2 clustering system, CRY2clust, by discovering a short peptide. We also discovered a role of the intrinsic C-terminal region of CRY2, suggesting the potential use of the C-terminal region of CRY2 for oligomerization. Through the serial deletion experiment and mutational analysis of the sequences of nine residues and intrinsic CRY2 sequences, we suggest that both CRY2clust and CRY2 [1–507] might have a similar mechanism to enhance CRY2 clustering; hydrophobicity of residue at a specific position is critical to determine light-induced CRY2 clustering. In addition, from the results in the co-translocation assay and InCell SMART-i assay (Supplementary Fig. 16), which visualizes specific protein interactions^25^, we suggest that CRY2clust and CRY2 [1–507] are not significantly involved in change of oligomeric state in the dark. The mechanism of increased clustering efficiency of CRY2clust and CRY2 [1–507] might be related to stabilization of activated CRY2PHR structure^17^, but further studies will be needed for precise explanation.

Compare to the previously developed CRY2 oligomerization modules, such as CRY2PHR and CRY2olig, the advantages of our system are fast dynamics for assembly and disassembly, higher sensitivity for light and distribution of clusters without accumulation in subcellular compartments such as nuclear speckles. The rapid clustering of CRY2clust improved the dynamics of previously reported CRY2-based optogenetic modules under the same condition of light illumination. Therefore, we conclude that our system has a high potential for versatile application with a great temporal resolution without perturbing target signaling in the dark, allowing to manipulate relatively rapid cellular responses.

In summary, our optogenetic clustering module enables rapid and efficient homo-oligomerization of proteins, which allow to control and investigate rapid signaling dynamics in vitro and in vivo. Moreover, our novel findings, such as critical determinants for efficient CRY2 clustering and a role of the intrinsic C-terminal region of CRY2, will contribute to better understanding of the mechanisms underlying light-induced CRY2 homo-oligomerization and higher-ordered cluster formation. We expect that our improved CRY2 module will expand the versatility of the optogenetic clustering toolbox and enable users to design the precise method for their specific experimental purposes.

## Methods

### Plasmid construction

Fluorescent protein (FP) expression plasmids for pECFP-C1, pmCerulean-C1, pEGFP-C1, pEYFP-C1, pmCitrine-C1, pDsRed-Express2-C1, and pmCherry-C1 were obtained from Clontech. Expression plasmids for pCypet-C1 (Addgene plasmid #54649, a gift from Patrick Daugherty and Michael Davidson), pYpet-C1 (Addgene plasmid #54648, a gift from Patrick Daugherty and Michael Davidson), FusionRed-C1 (Addgene plasmid #54777, a gift from Michael Davidson)^26^, mRFP-C1 (Addgene plasmid #54764, a gift from Robert Campbell, Michael Davidson, and Roger Tsien) and R-GECO1 (Addgene plasmid #32444, a gift from Robert Campbell)^22^ were obtained from Addgene. *dTomato*, *tdTomato*, or *Ruby 2* cDNA from paavCAG-post-mGRASP-2A-dTomato (Addgene plasmid #34912, a gift from Jinhyun Kim)^27^, pCSCMV:tdTomato (Addgene plasmid #30530, a gift from GerhartRyffel)^28^ and pcDNA3-mRuby2 (Addgene plasmid #40260, a gift from Michael Lin)^29^, respectively, flanked by *Age*I and *Bsr*GI restriction sites, were amplified by polymerase chain reaction (PCR) and inserted into pECFP-C1 after excision of *ECFP* by digestion with AgeI and BsrGI, to generate dTomato-C1, tdTomato-C1 and Ruby2-C1 vectors. Expression plasmids for FPs-CRY2PHR and CRY2PHR-FPs were constructed by insertion of sequences encoding codon-optimized CRY2PHR (amino acids 1–498)^10^ into *Nhe*I and *Age*I sites or *Eco*RI, and *Bam*HI sites of each FP vector. Sequences encoding CRY2 fragments (amino acids 1–499, 1–500, 1–501, 1–502, 1–503, 1–504, 1–505, 1–506, and 1–507) were PCR-amplified and inserted into pmCherry-CRY2PHR at *Eco*RI and *Bam*HI sites to create mCherry-CRY2 variants. The sequence encoding CRY2clust from pmCherry-CRY2clust was PCR-amplified using the primer pair 5′-GTA GCT AGC CAC CAT GAA GAT GGA CAA AAA GAC CAT CGT CTG-3′ (forward) and 5′-GAC TCT CGA GTG AAC CTG AAC CTG AAC CTG AAC CGT TGT CGA GGT CGG GGG GGT CC-3′ (reverse) and inserted into pmCherry-N1 (Clontech) at *Nhe*I and *Xho*I sites to generate the CRY2clust-mCherry plasmid.mCherry-CRY2clust variants, CRY2clust mutants, and CRY2 [1–507] mutants were constructed by first changing the nucleotides encoding Arg at position 489 of CRY2PHR from CGG to CGA, generating a *Xho*I site, and then inserting oligonucleotides encoding the C-terminus of CRY2PHR (amino acids 489–498) and CRY2clust variants or mutants, or the C-terminus of CRY2 (amino acids 489–506) and F507X mutants into the vector at *Xho*I and *Bam*HI sites. The sequence encoding CRY2PHR was mutagenized through PCR-driven overlap extension and inserted into mCherry-C1 at *Eco*RI and *Bam*HI sites to generate mCherry-CRY2olig expression vectors. The EGFP-SC35 expression vector was generated by inserting *SC35*cDNA from pcDNA3.1-SC35-cMyc (Addgene plasmid #44721, a gift from Kathleen Scotto)^30^ into pEGFP-C2 (Clontech) at *Eco*RI and *Bam*HI sites. For construction of EGFP-CRY2clust-linker-STIM1 (OptoSTIM1(CRY2clust)), sequences encoding CRY2clust from pmCherry-CRY2clust were PCR-amplified and inserted into the previously constructed EGFP-Cry2-linker-STIM1 (OptoSTIM1)^7^ at *Age*I and *Bsr*GI sites after excising the *Cry2* gene from the vector. Sequences encoding Raf1 were PCR-amplified and inserted into mCherry-CRY2PHR or mCherry-CRY2clust at *Xho*I and *Hin*dIII sites by using Gibson Assembly Master Mix (NEB) to create mCherry-Raf1-CRY2PHR or mCherry-Raf1-CRY2clust expression vector, respectively. The mCerulean-Raf1-CRY2PHR and mCerulean-Raf1-CRY2clust were generated by replacing *mCherry*cDNA in mCherry-Raf1-CRY2PHR and mCherry-Raf1-CRY2clust expression vectors, respectively, with mCerulean from pmCerulean-C1 (Clontech) by *Nhe*I and *Bsr*GI sites. For construction of ERK2-mEGFP, sequences encoding ERK2 from pEX_EF1_ERK2-YFP (Alliance for Cellular Signaling) were PCR-amplified and inserted into mEGFP-N1 (Addgene plasmid # 54767, a gift from Michael Davidson) at *Xho*I and *Xma*I sites. The ERK KTR-FusionRed expression vector was constructed by first PCR-amplifying sequences encoding either Elk1 or FusionRed from pLentiCMVPuro DEST ERKKTRClover (Addgene plasmid # 59150, a gift from Markus Covert)^24^ or FusionRed-C1, respectively, and overlap extension PCR-amplifying with these two PCR-amplified fragments, and then ligating them into pLentiCMVPuro DEST ERKKTRClover at *Bsr*GI site after excising both *Elk1* and *clover* genes from the vector.

### Cell culture and transfection

HeLa cells (obtained from American Type Culture Collection) were maintained in Dulbecco’s Modified Eagle’s Medium (Gibco) supplemented with 10% fetal bovine serum (Invitrogen) at 37 °C in a humidified 10% CO_2_ atmosphere. Cells were confirmed to be free from mycoplasma using an e-Myco Mycoplasma PCR detection kit (iNtRON). For live-cell imaging, cells were plated on a 96-well plate (μ-Plate 96 Well ibiTreat; ibidi GmbH) and transfected using either a Microporator (Neon Transfection System; Invitrogen) or Lipofectamine LTX (Invitrogen), according to the manufacturers’ instructions. For HeLa cells, electroporation conditions (two pulses of 980 V for 35 ms) were additionally optimized for higher transfection efficiency.

### Live-cell imaging

Live-cell imaging was performed using a Nikon A1R confocal microscope (Nikon Instruments) mounted onto a Nikon Eclipse Ti body equipped with a Nikon CFI Plan Apochromat VC objective (60×/1.4 numerical aperture [NA]; Nikon Instruments) and digital-zooming Nikon imaging software (NIS-element AR 64-bit version 3.21; Laboratory Imaging). A Chamlide TC system placed on a microscope stage was used for maintaining environmental conditions at 37 °C and 10% CO_2_ (Live Cell Instruments). Photoexcitation was delivered using a photostimulation module in Nikon imaging software (NIS-elements) that provided one loop of 1 s stimulation with a 488-nm laser. A light power density of 490 μW mm^−2^ (measured with an optical power meter from ADCMT) was used for photoexcitation, unless stated otherwise.

### Structured illumination microscopy (SIM) imaging

HeLa cells coexpressing mCherry-labeled CRY2olig or CRY2clust with EGFP-SC35 were light illuminated for 5 min using a TouchBright W-96 LED Excitation System (Live Cell Instrument); under our experimental conditions, LED power density corresponded to 3 mW cm^−2^ (at 470 nm). Cells were immediately fixed with a mixture of 3% paraformaldehyde and 0.1% glutaraldehyde in phosphate-buffered saline (PBS) at room temperature for 5 min. After washing three times with PBS, fixed cells were imaged using a Nikon Structured Illumination Microscope (N-SIM; Nikon Instruments) equipped with a CFI SR Apochromat TIRF objective (100×/1.49 NA; Nikon Instruments).

### Imaging processing and analysis

Images were analyzed with Nikon imaging software (NIS-elements AR 64-bit version 3.21; Laboratory Imaging), MetaMorph software (version 7.8.1.0; MDS Analytical Technologies) or ImageJ software (version 1.50b; U.S. National Institutes of Health; http://imagej.nih.gov/ij/). For quantification of cluster formation, clusters were defined as discrete puncta of fluorescence with criteria of fluorescence intensity (1,500–4,095 arbitrary units) and equivalent diameter (>0.2 μm). The percentage of clustered cells was calculated by dividing the number of cells that formed clusters upon blue light stimulation by the total population of transfected cells. The cluster ratio in cells was quantified using “Automated Measurement” and “ROI Statistics” tools in Nikon imaging software. The total cluster intensity (*I*_C_) was divided by the total fluorescence intensity (*I*_W_) of the whole cell. Cluster size distribution was determined by classifying different cluster areas using the “Annotated Measurement Results” in Nikon imaging software. Co-localization between two molecules with different fluorescent colors was quantified with Pearson’s correlation coefficient by using the “Co-localization” tool in Nikon imaging software. A “Kymograph” tool in MetaMorph software was used to draw kymographs. Statistical significance was evaluated using a two-tailed Student’s *t*-test.

### Data availability

All data generated or analyzed during this study are included in this published article and in Supplementary Information files. They are available from the corresponding author upon reasonable request.

## Electronic supplementary material

Supplementary Information

Supplementary Movie 1

Supplementary Movie 2
