# Supplementary material for: Optogenetic protein clustering through fluorescent protein tagging and extension of CRY2
Source: Nat Commun. 2017 Jun 23;8:30. doi: 10.1038/s41467-017-00060-2 (PMC5482817; doi:10.1038/s41467-017-00060-2)
Supplement: Supplementary file 1 — Supplementary Information [file 41467_2017_60_MOESM1_ESM.pdf]

File Name: Supplementary Information
Description: Supplementary Figures

File Name: Supplementary Movie 1
Description: Cluster formation of CRY2PHR conjugated with various fluorescent proteins upon blue light illumination. Fluorescence images of HeLa cells expressing each FP-labeled CRY2PHR were captured at 10 s intervals for 5 min. Numbers indicate minutes:seconds.

File Name: Supplementary Movie 2
Description: Rapid and reversible clustering of CRY2clust. Fluorescent images of HeLa cell expressing mCherry-CRY2clust were captured at 10 s intervals for 18 min. After 1 min imaging, a cell was illuminated by blue light for 1 s. Numbers indicate minutes:seconds.

Supplementary Figure 1. Diverse efficiency of CRY2 clustering by conjugating different fluorescent proteins.

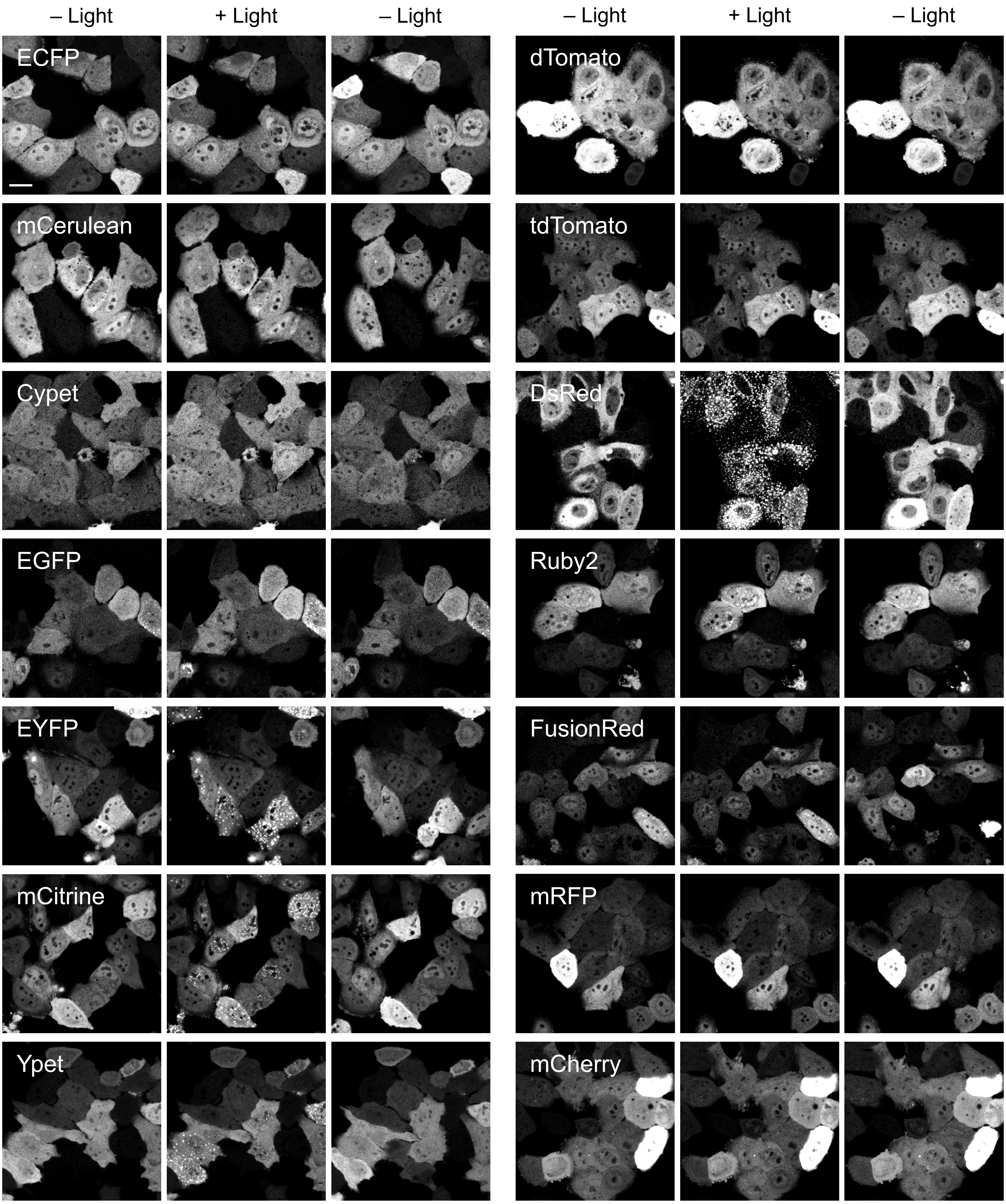

HeLa cells expressing each CRY2 construct conjugated with the indicated fluorescent protein were illuminated by blue light for 10 min at 20 s intervals. Scale bar, 20 μm.

Supplementary Figure 2. Effect of conjugation site of fluorescent protein (FP) on the efficiency of CRY2 clustering.

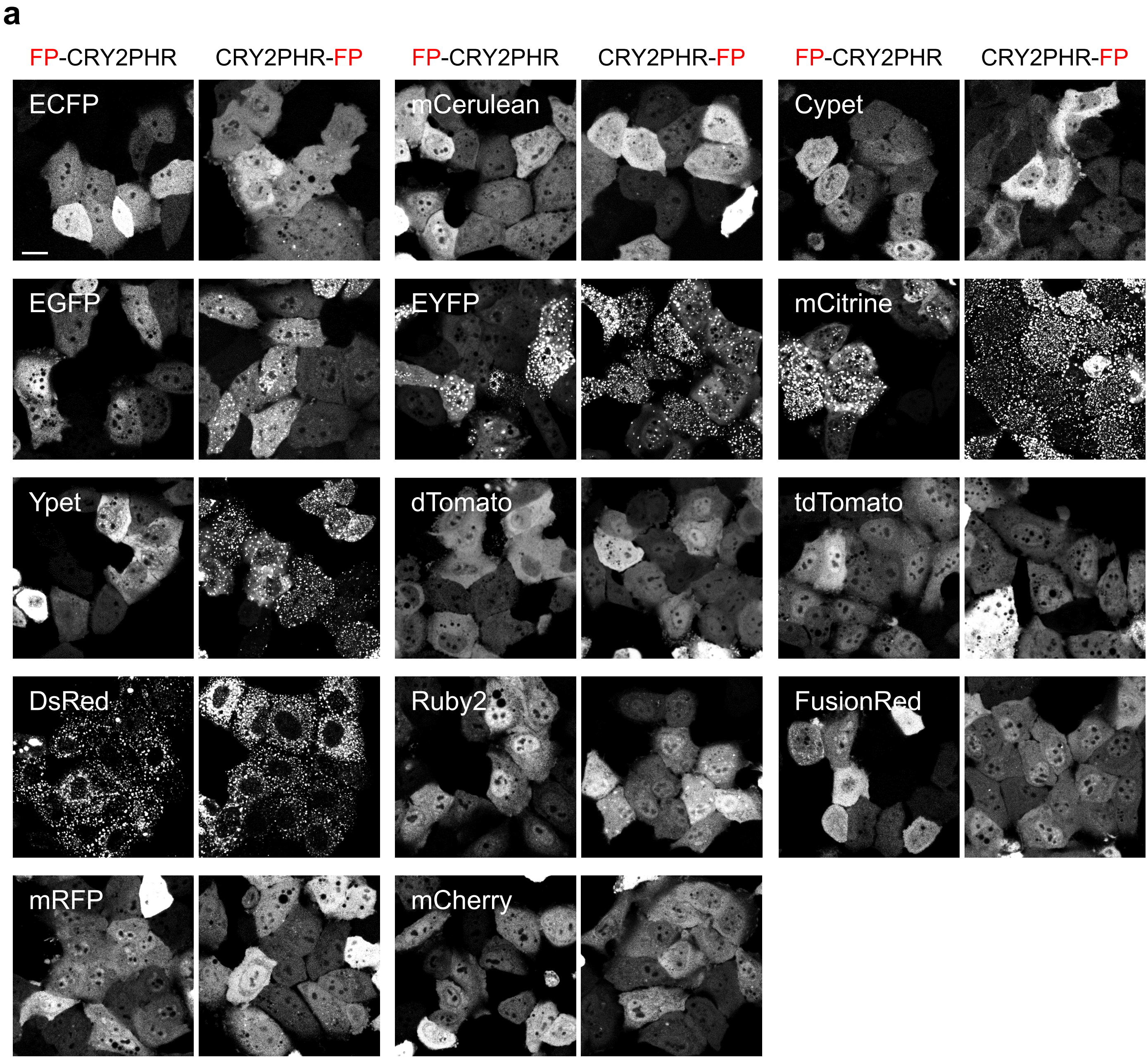

b

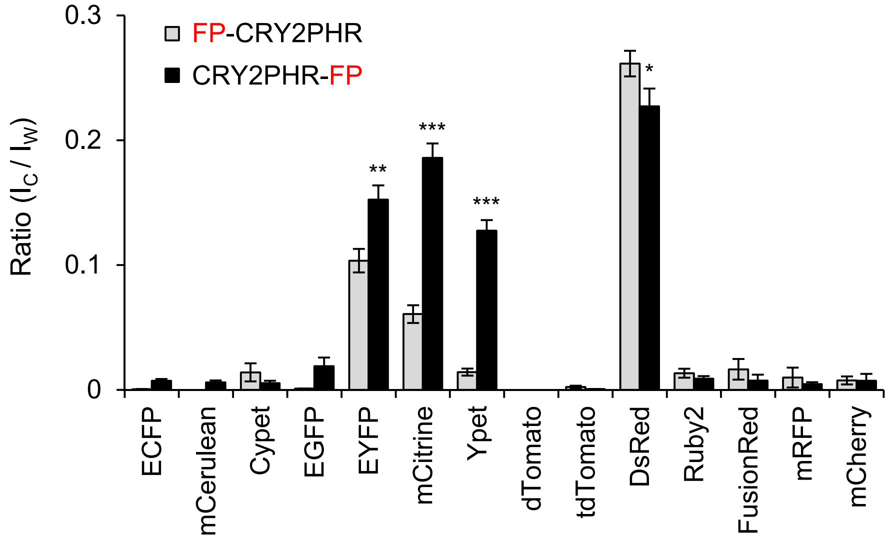

(a) Fluorescence images of HeLa cells expressing the indicated fluorescent protein construct conjugated to either the N-terminus or C-terminus of CRY2PHR. Cells were exposed to blue light for 5 min at 20 s intervals. Scale bar, 20 μm. **(b)** Quantification of cluster ratio in cells containing clusters. For quantification, total cluster intensity (I_C) was divided by the total fluorescence intensity (I_W) of the whole cell. Values are expressed as means ± s.e.m. **P* = 0.04; ***P* = 0.0009; ****P* = 5.89×10^{-16} (mCitrine), 1.58×10^{-24} (Ypet) by Student's two-tailed *t*-test.

Supplementary Figure 3. Effect of the peptide conjugated to either the C-terminus or N-terminus of CRY2PHR on its clustering.

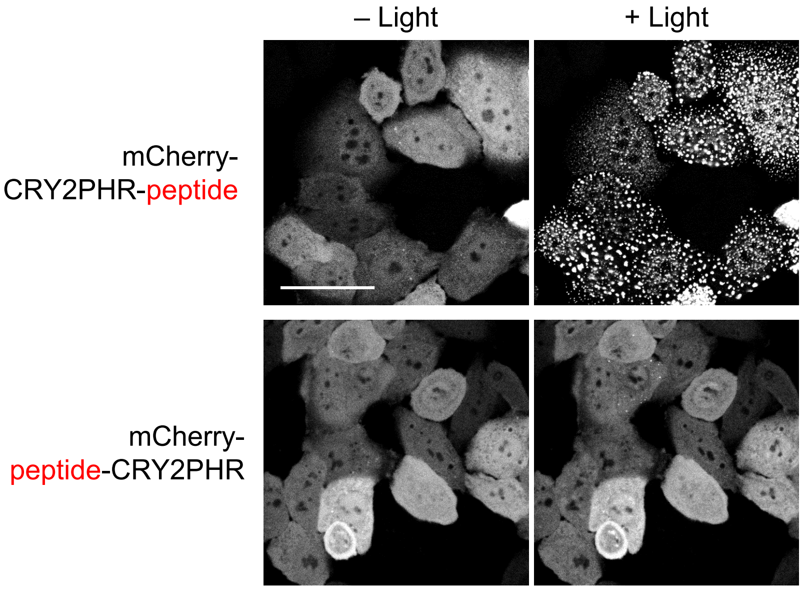

Fluorescence images of HeLa cells expressing the mCherry-labeled CRY2PHR constructs conjugating with the 9-residue peptide at either the C-terminus or N-terminus of CRY2PHR. Cells were exposed to blue light for 5 min at 20 s intervals. Scale bar, 50 µm.

Supplementary Figure 4. Light-induced clustering of CRY2clust variants with C-terminal serial deletions.

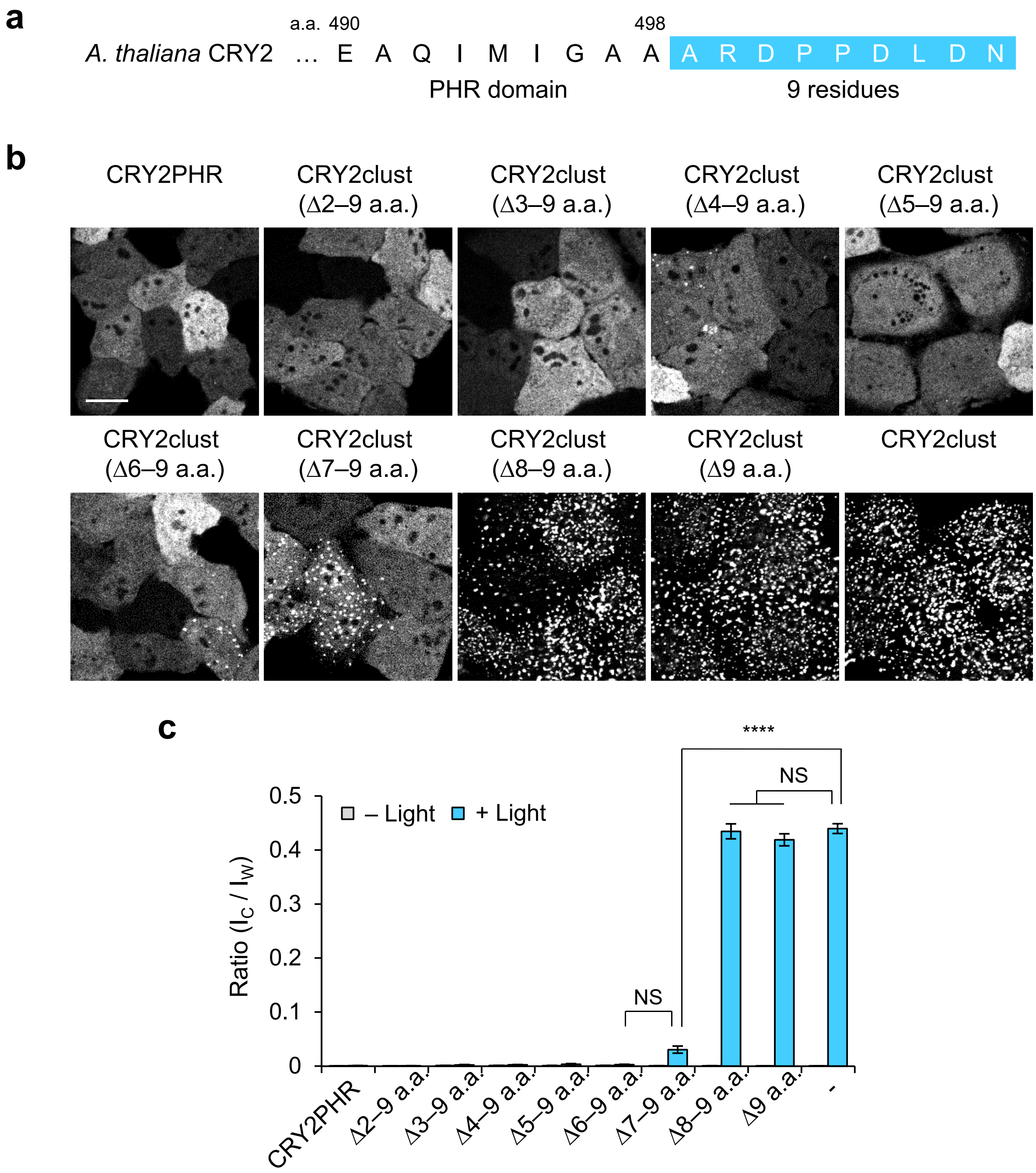

(a) Sequence of the C-terminus of CRY2clust. The 9-residue of peptide are shown in the blue box. **(b)** Fluorescence images of HeLa cells expressing the indicated mCherry-labeled CRY2clust variants with its C-terminal serial deletions. Cells were exposed to light for 10 min at 20 s intervals. Scale bar, 20 μm. **(c)** Quantification of cluster ratio showing that residues 7 to 9 of the C-terminal peptide in CRY2clust are important for enhancing light-induced CRY2 clustering. Ic, total cluster intensity; I_W, total whole-cell intensity. Values are expressed as means ± s.e.m. ****$P = 1.48 \times 10^{-91}$; NS, not significant by Student's two-tailed t-test.

Supplementary Figure 5. The extent of homo-association of CRY2PHR or CRY2clust in dark state.

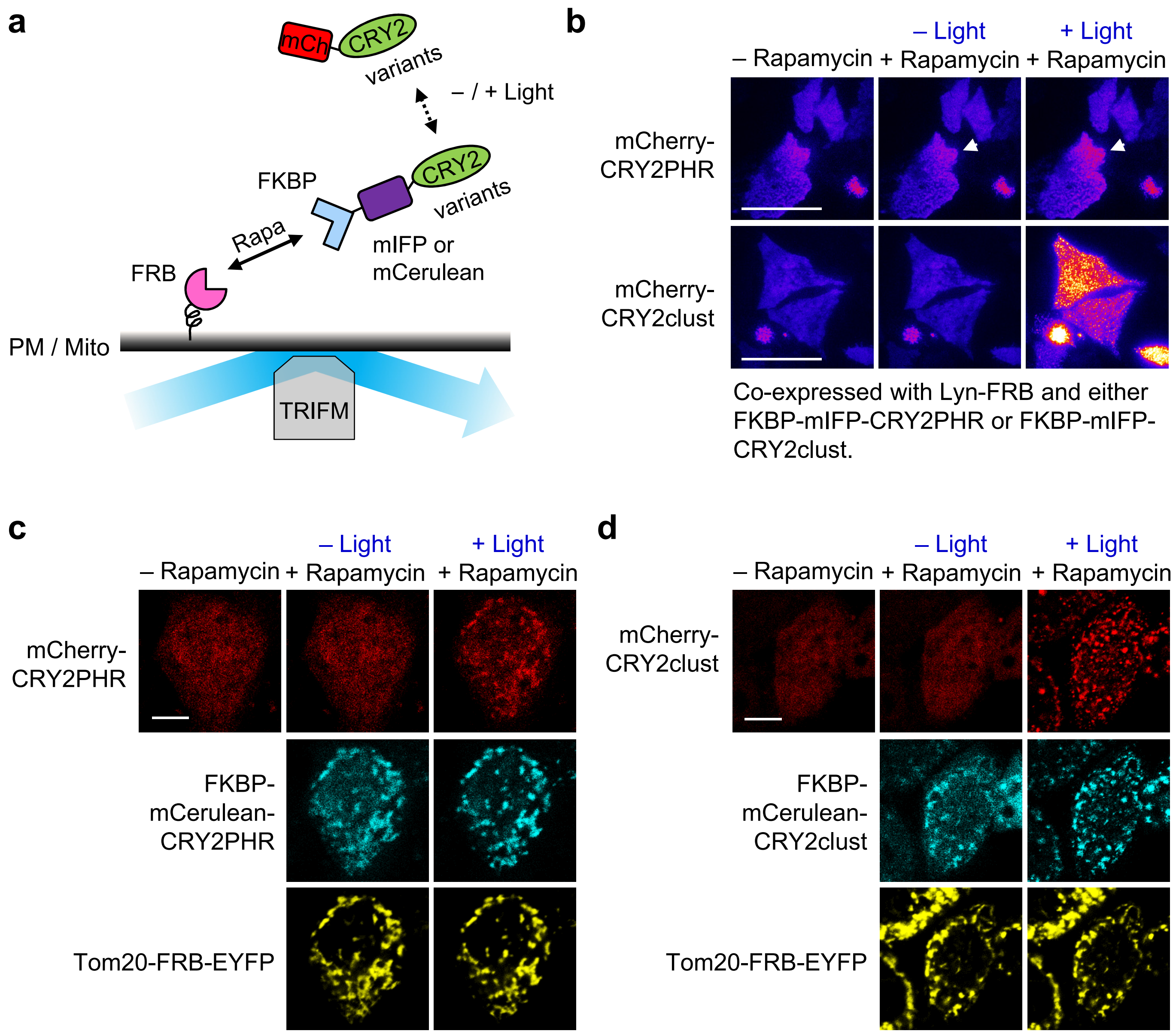

(a) Schematic diagram for translocation assay of CRY2PHR variants to plasma membrane (PM) or mitochondria (Mito) with orthogonal inputs: rapamycin and light. **(b)** Total internal reflection fluorescence microscopy (TIRFM) images of HeLa cells co-expressing Lyn-FRB, FKBP-mIFP-CRY2PHR variants (CRY2PHR and CRY2clust), and mCherry-CRY2PHR variants before and after treatment of rapamycin and light illumination. Scale bar, 50 μm. **(c-d)** Fluorescence images of translocation of CRY2PHR (**c**) and CRY2clust (**d**) to the mitochondria before and after treatment of rapamycin and light illumination.

Supplementary Figure 6. Importance of hydrophobicity of Leu at position 7 in the C-terminal peptide of CRY2clust for light-induced CRY2 clustering.

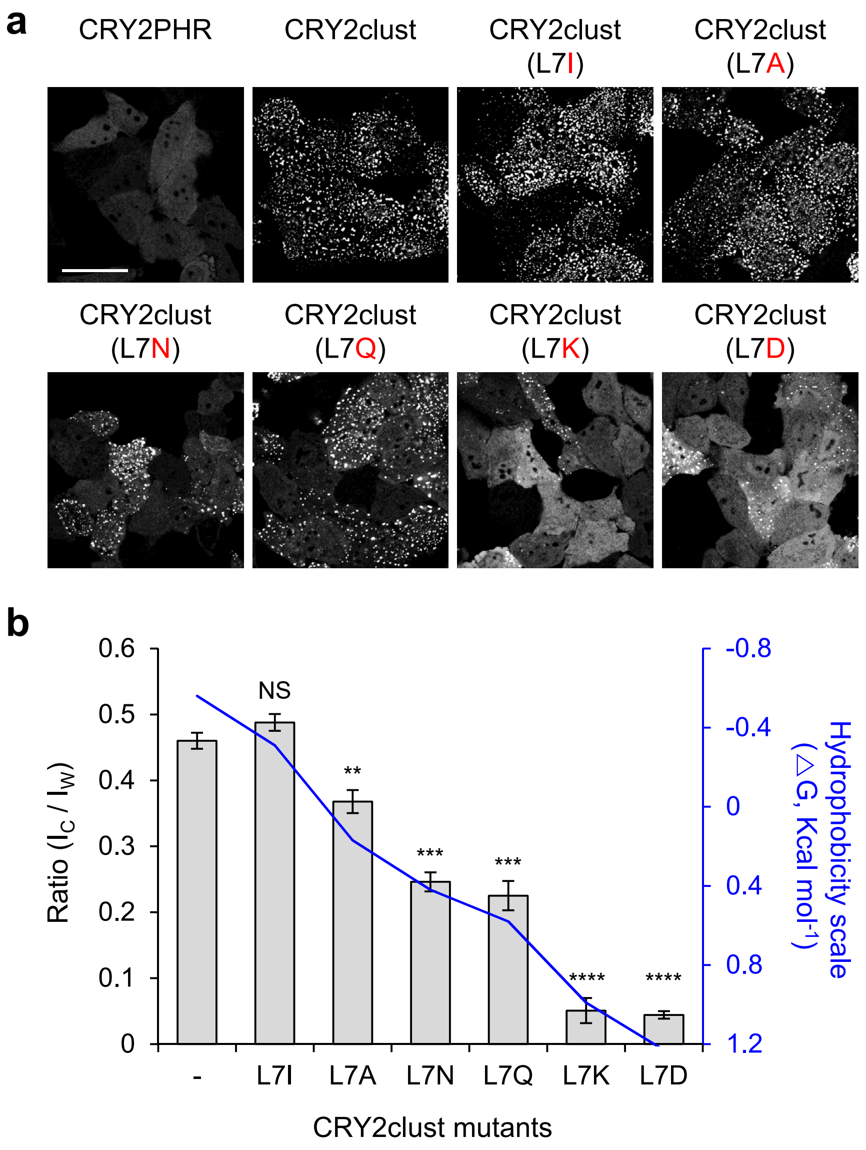

(a) Fluorescence images showing clustering of CRY2clust mutants, upon blue light illumination. HeLa cells expressing the indicated mCherry-labeled CRY2clust mutants were illuminated by blue light for 10 min at 20 s intervals. Scale bar, 50 μm. **(b)** Combinatorial graph representing cluster ratio of CRY2clust mutants (gray boxes) and hydrophobicity scale of substitutes (blue line). Ic, total cluster intensity; I_W, total whole-cell intensity. Values are expressed as means ± s.e.m. $**P = 1.69 \times 10^{-6}$ (L7A); $***P = 2.78 \times 10^{-14}$ (L7N), 3.87×10^{-20} (L7Q); $****P = 3.84 \times 10^{-118}$ (L7K), 8.37×10^{-121} (L7D); NS, not significant by Student's two-tailed *t*-test.

Supplementary Figure 7. Light-induced clustering of CRY2 extended with the intrinsic CRY2 sequence at the C-terminus of CRY2PHR domain.

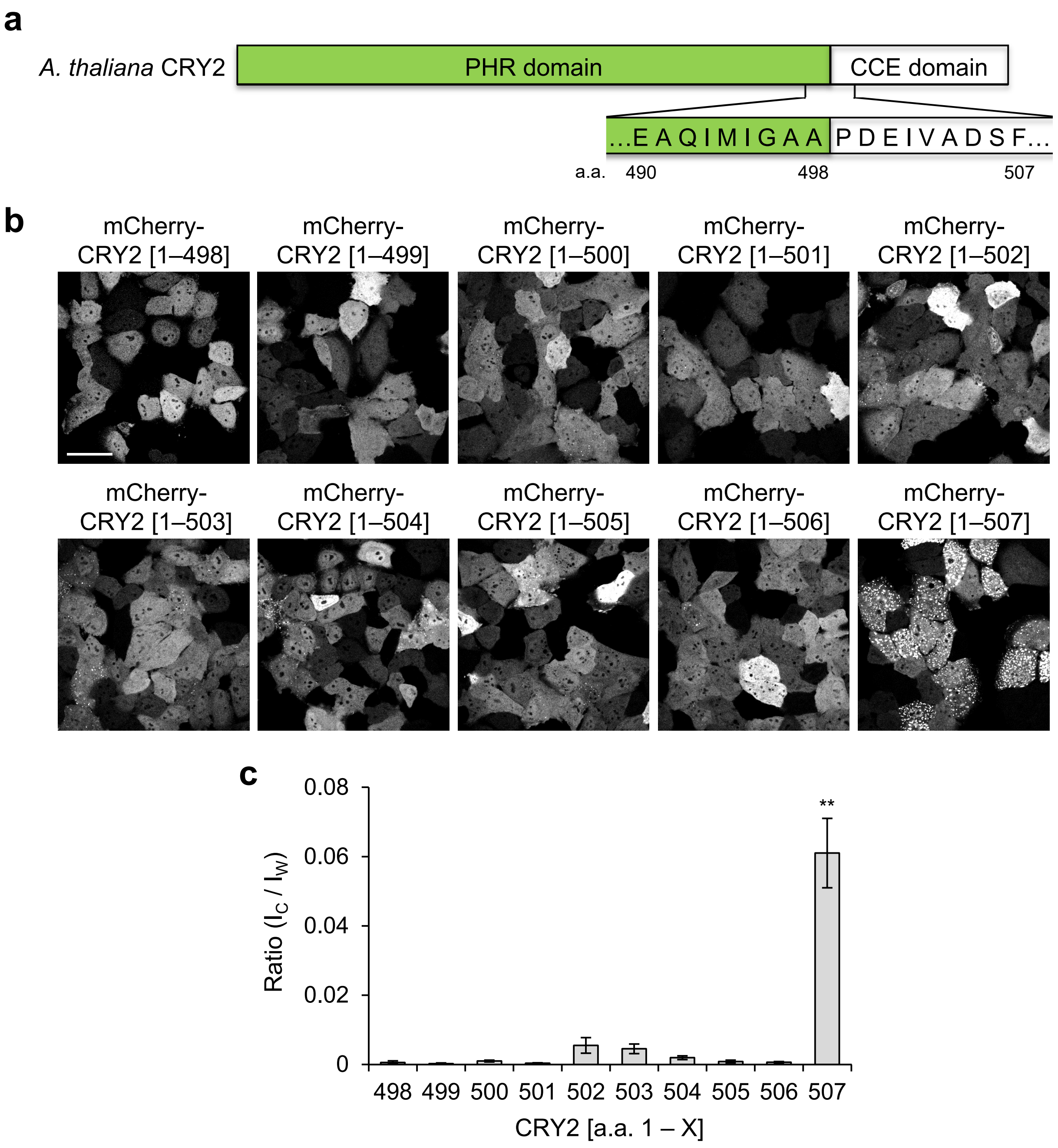

(a) *A. thaliana* CRY2 amino acid sequence showing the PHR domain (green box) and CCE (Cryptochrome C-terminal Extension) domain. **(b)** Fluorescence images showing clustering of CRY2 variants, extended serially with the native CRY2 sequence at the C-terminus of the PHR domain, upon blue light illumination. HeLa cells expressing the indicated variants were illuminated by blue light for 10 min at 20 s intervals. Scale bar, 50 μm. **(c)** Quantification of cluster ratio showing that the 9-amino-acid extension of the C-terminus of the PHR domain dramatically enhances light-induced CRY2 clustering. Ic, total cluster intensity; I_W, total whole-cell intensity. Values are expressed as means ± s.e.m. $^{**}P = 3.08 \times 10^{-8}$ by Student's two-tailed *t*-test.

Supplementary Figure 8. Importance of hydrophobicity of Phe at position 507 for light-induced CRY2 clustering.

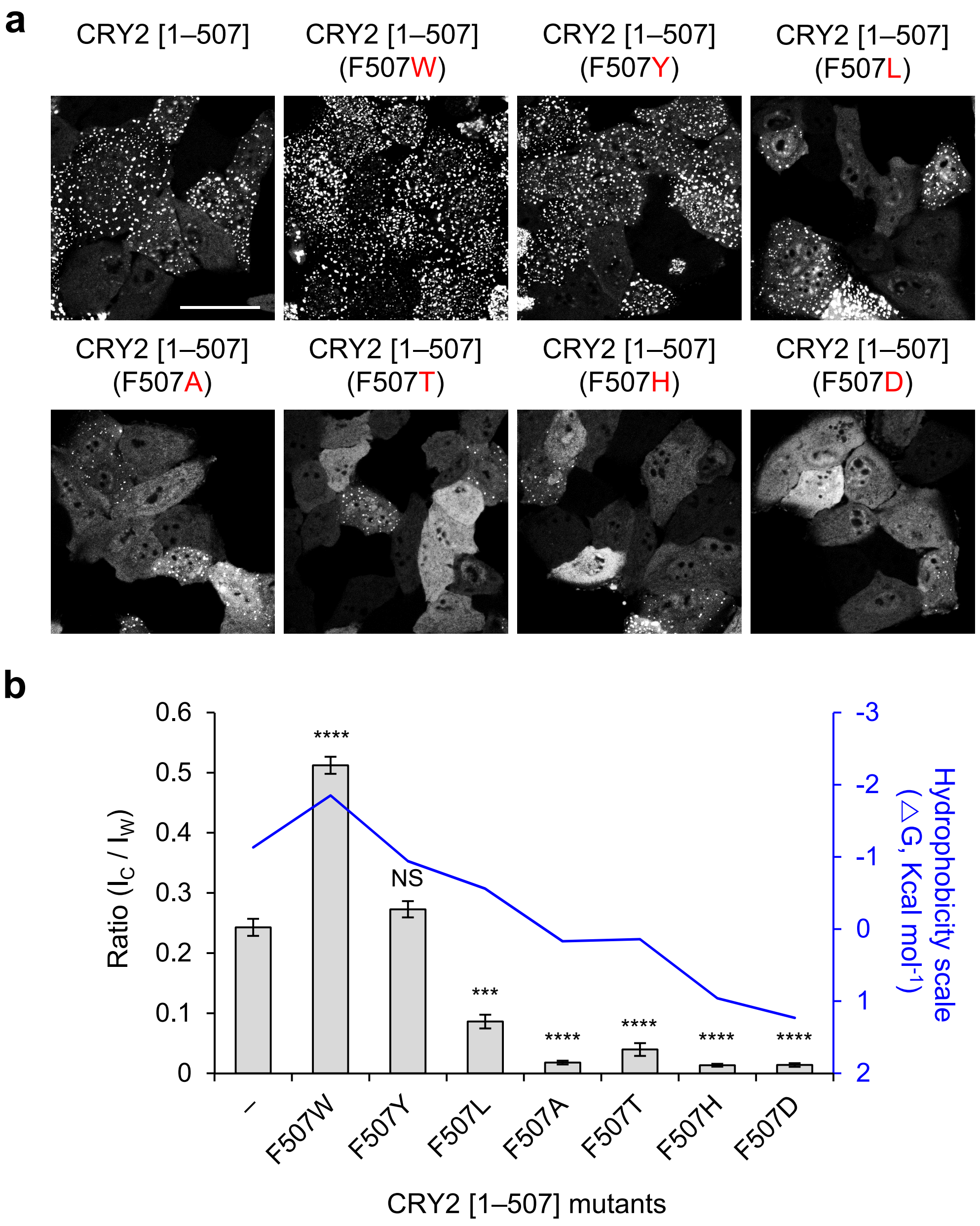

(a) Fluorescence images showing clustering of CRY2 [1–507] mutants, upon blue light illumination. HeLa cells expressing the indicated mCherry-labeled CRY2 [1–507] mutants were illuminated by blue light for 10 min at 20 s intervals. Scale bar, 50 μm. **(b)** Combinatorial graph representing cluster ratio of CRY2 [1–507] mutants (gray boxes) and hydrophobicity scale of substitutes (blue line). Ic, total cluster intensity; I_W, total whole-cell intensity. Values are expressed as means ± s.e.m. ****P* = 1.95 × 10^{-16} (F507L); *****P* = 3.87 × 10^{-30} (F507W), 5.18 × 10^{-37} (F507A), 4.06 × 10^{-25} (F507T), 4.2 × 10^{-37} (F507H), 5.95 × 10^{-38} (F507D); NS, not significant by Student's two-tailed *t*-test.

Supplementary Figure 9. Nuclear-localization pattern of clusters of CRY2olig and CRY2clust upon light stimulation.

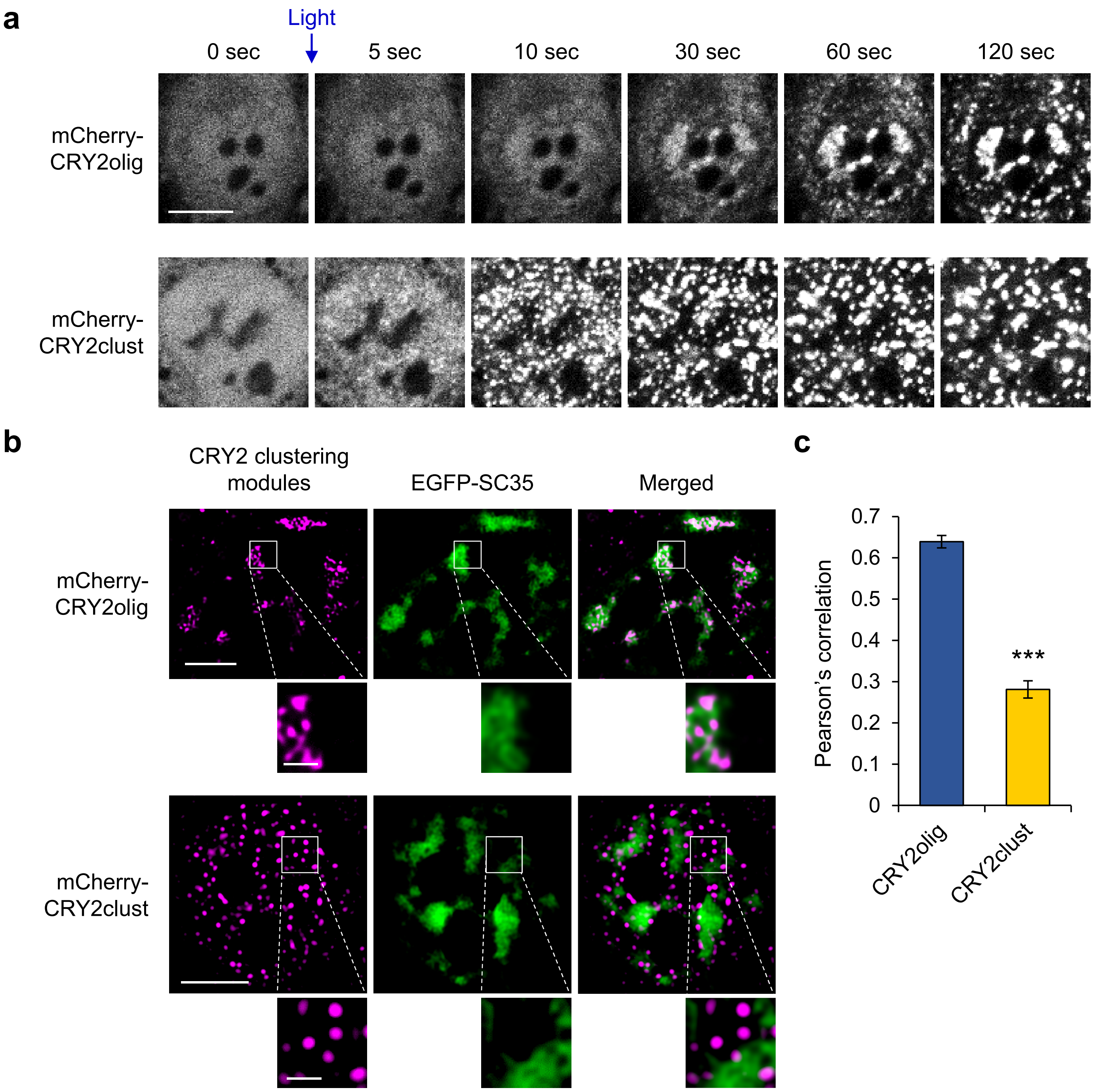

(a) Fluorescence images showing cluster distribution in the nucleus. HeLa cells transfected with expression plasmids for mCherry-CRY2olig or mCherry-CRY2clust were illuminated with blue light for 1 s. Blue arrow indicates time of light illumination. **(b)** Structured illumination microscopy (SIM) images of cluster patterns for each CRY2 clustering module in the nucleus. **(c)** Colocalization analysis between each CRY2 clustering module and EGFP-SC35. Values are expressed as means ± s.e.m. ***$P = 7.53 \times 10^{-16}$ by Student's two-tailed *t*-test. Scale bars, 10 μm (**a**), 5 μm (**b**), and 1 μm (**b**, insets).

Supplementary Figure 10. Correlation between $T_{1/2}$ and expression level of CRY2olig and CRY2clust.

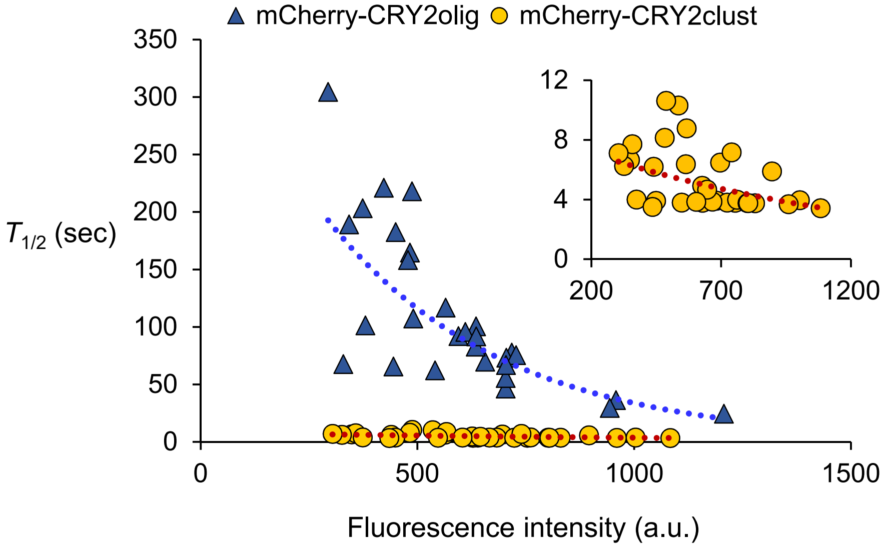

Scatter plot of the time required to reach half-maximal cluster ratio ($T_{1/2}$) of each cell based on the mCherry fluorescence intensity (a.u.) of the indicated CRY2 clustering modules. Dotted lines indicate trend lines. Although both the kinetics for clustering of CRY2olig and CRY2clust (inset graph) depend on expression level, the CRY2clust showed much faster kinetics than CRY2olig for cluster formation at even low concentration.

Supplementary Figure 11. Correlation between cluster efficiency and expression level of CRY2 clustering modules.

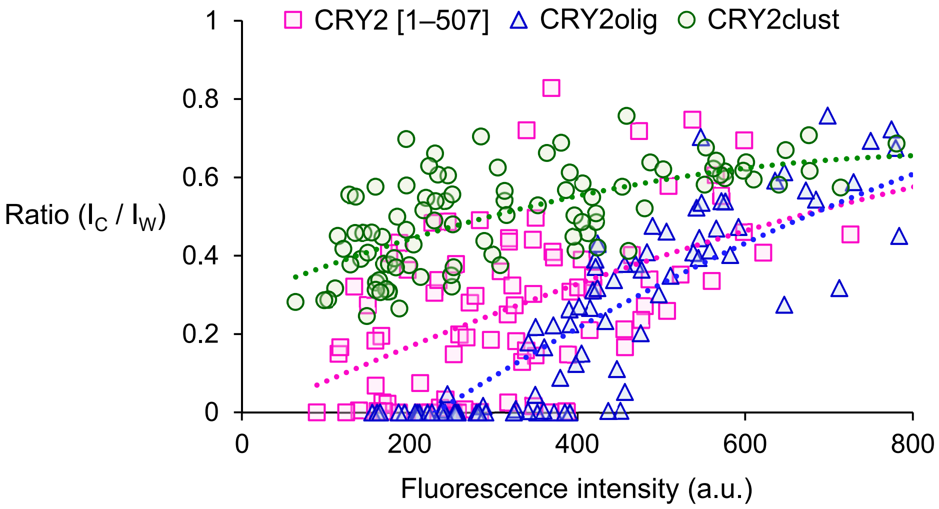

Scatter plot of cluster ratio of each cell based on the mCherry fluorescence intensity (a.u.) of the mCherry-labeled indicated CRY2 clustering modules. Dotted lines indicate trend lines. The cluster efficiency showed the positive correlation with expression level in all CRY2 clustering modules. Ic, total cluster intensity; I_W, total whole-cell intensity.

Supplementary Figure 12. Comparative light-sensitivity of CRY2olig and CRY2clust at different laser powers.

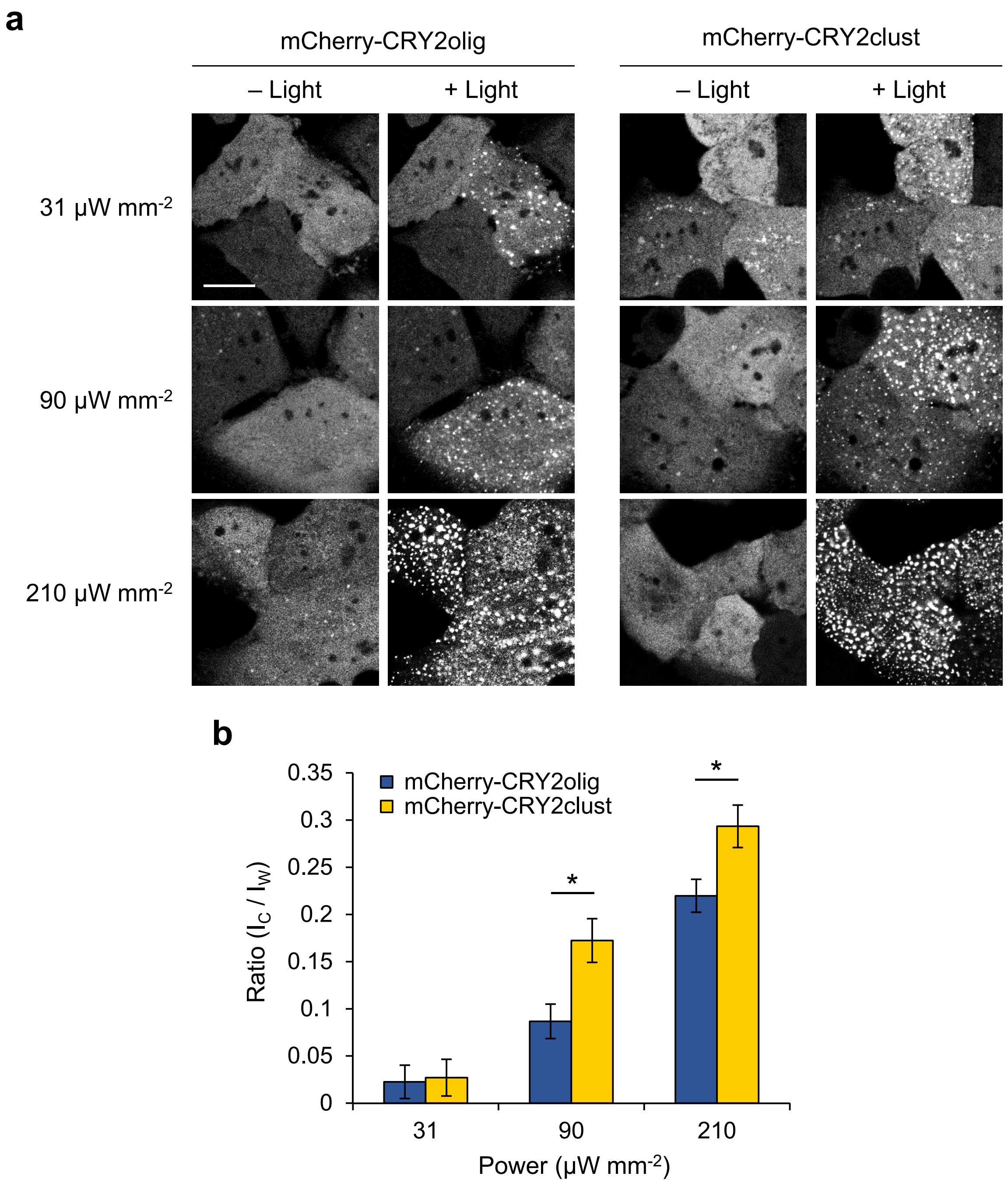

(a) Fluorescence images showing cluster formation at different power densities of 488-nm laser light in HeLa cells expressing either mCherry-CRY2olig or mCherry-CRY2clust. Scale bar, 20 μm. **(b)** Quantification of cluster ratio according to power densities shown in **a**. Ic, total cluster intensity; I_W, total whole-cell intensity. Values are expressed as means ± s.e.m. *P = 0.003 (90 μW mm^{-2}), 0.01 (210 μW mm^{-2}) by Student's two-tailed *t*-test.

Supplementary Figure 13. Effect of conjugation site of target protein on enhanced CRY2 clustering.

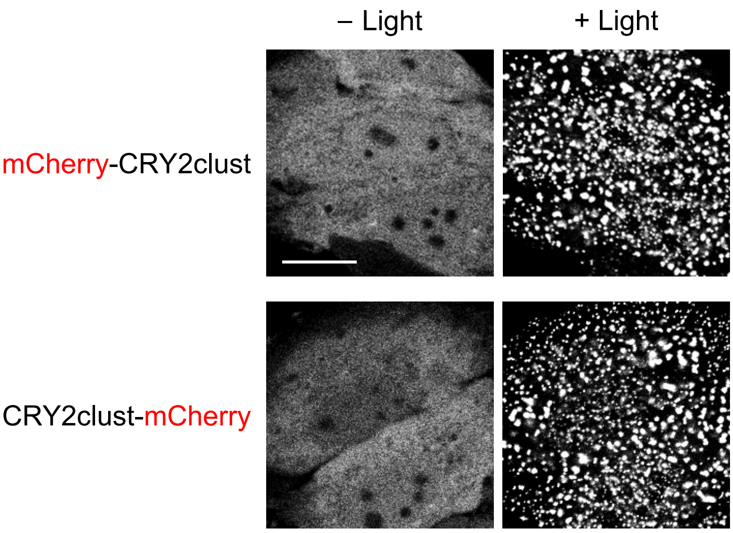

HeLa cells transfected with expression plasmids for mCherry-CRY2clust or CRY2clust-mCherry were illuminated with blue light for 5 min at 20 s intervals. Scale bar, 20 µm.

Supplementary Figure 14. Kinetics and efficacy of Ca^{2+} influx by OptoSTIM1 variants applied different CRY2 clustering modules.

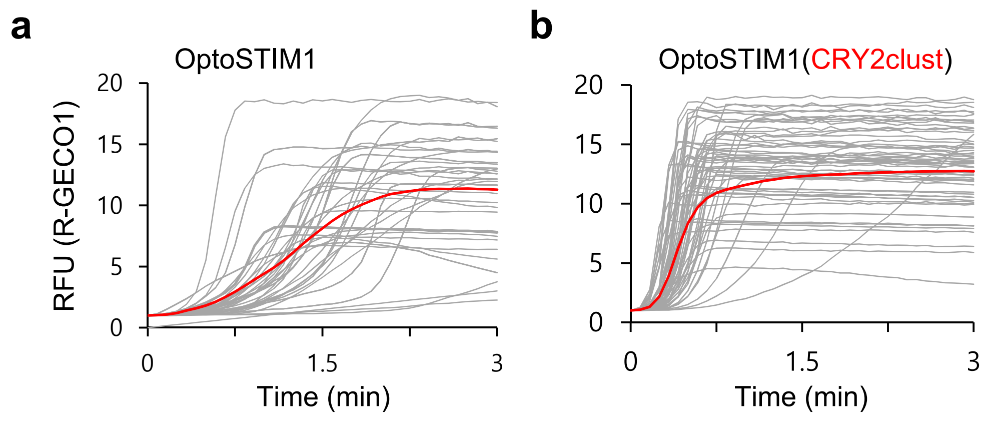

Time-lapse graphs representing changes of R-GECO1 fluorescence intensity by **(a)** original OptoSTIM1 and **(b)** OptoSTIM1(CRY2clust). Gray lines indicate the change in each individual cell. Red lines represent average values of R-GECO1 fluorescence intensity.

Supplementary Figure 15. Improved optogenetic activation of Raf1 signaling by applying CRY2clust.

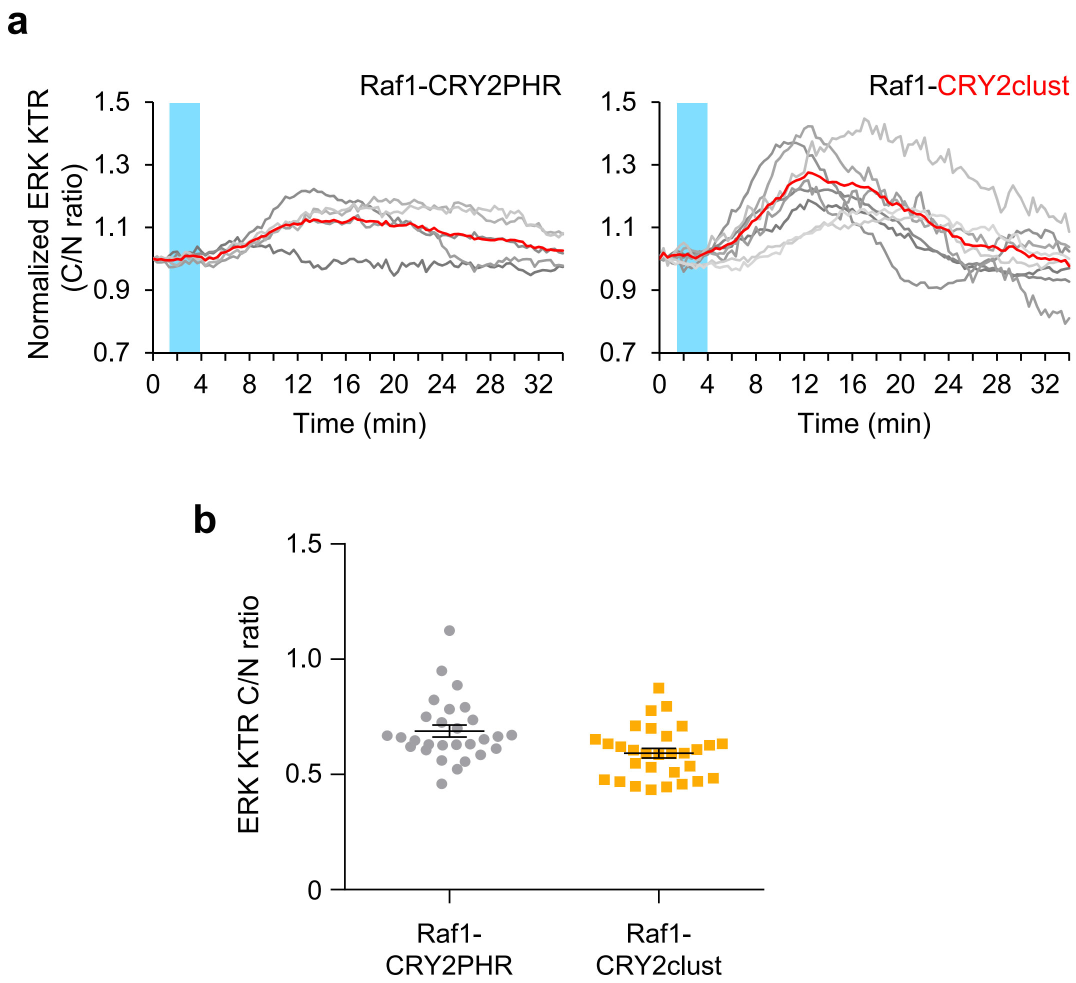

(a) Quantification of normalized translocation of ERK KTR sensor in HeLa cells coexpressing ERK KTR-FusionRed with either mCerulean-labeled Raf1-CRY2PHR or Raf1-CRY2clust. For quantification, fluorescence intensity of cytosolic ERK KTR was divided by the fluorescence intensity of the nuclear ERK KTR. Blue lines indicate illumination time points. Gray lines indicate translocation kinetics in each individual cell. Red lines indicate the averaged translocation kinetics. **(b)** Graphs showing basal ERK KTR C/N ratio of each cell plotted as a gray circle (Raf1-CRY2PHR) or a yellow box (Raf1-CRY2clust). Black lines are expressed as means ± s.e.m.

Supplementary Figure 16. Examination of homo-interaction of CRY2PHR variants in the dark.

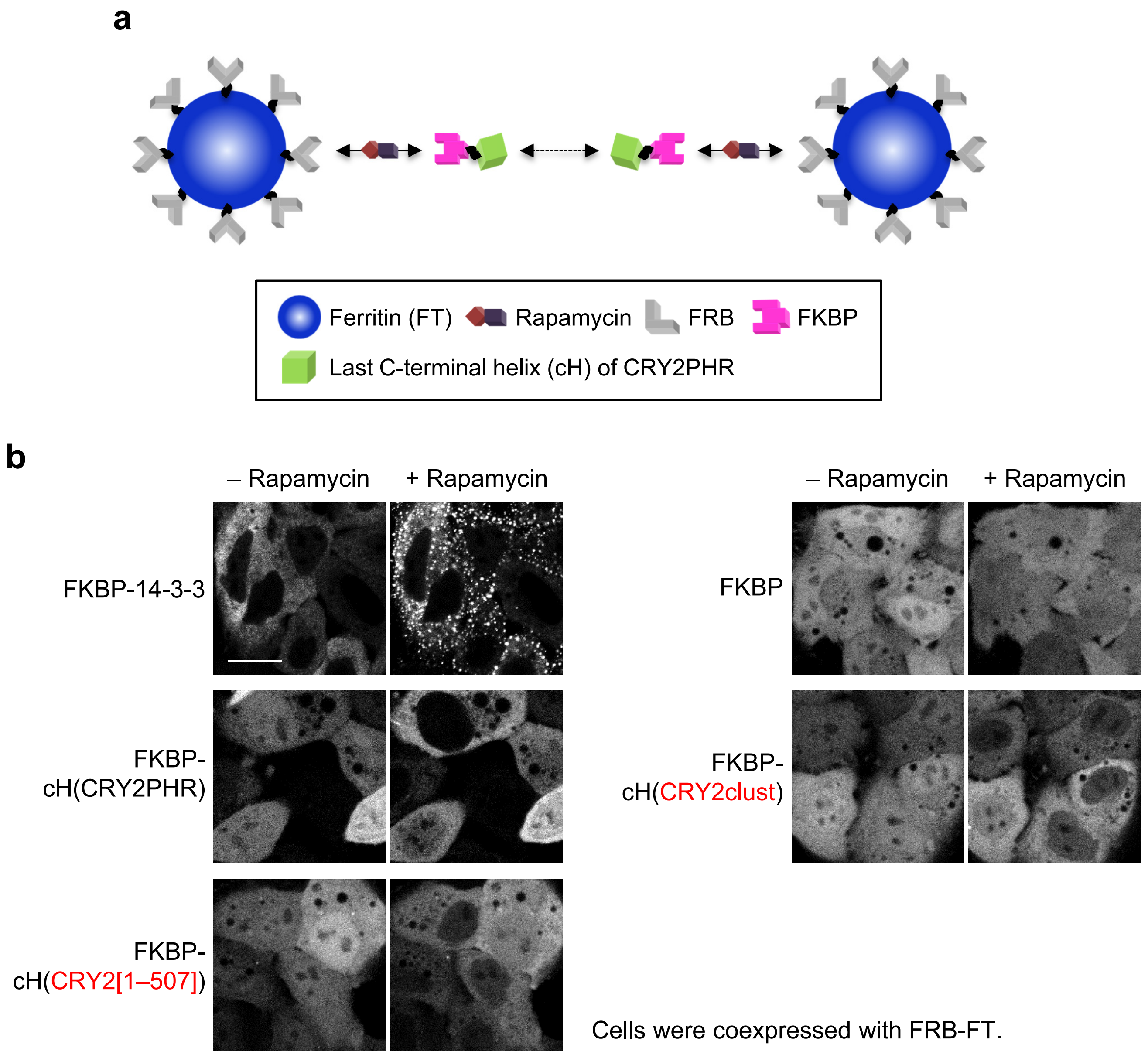

(a) InCell SMART-i assay for visualizing homo-association of CRY2PHR variants inside intracellular environment in the dark. (**b**) Fluorescence images of HeLa cells coexpressing indicated FKBP-fused construct with FRB-FT as indicated. Scale bar, 20 μm.
